# Study of THz-Plasmon hybridization of a loop Yagi-Uda absorber

**DOI:** 10.1038/s41598-017-17311-3

**Published:** 2017-12-05

**Authors:** Arnab Pattanayak, Sandipta Roy, Goutam Rana, Siddhartha P. Duttagupta, Venu Gopal Achanta, S. S. Prabhu

**Affiliations:** 1 Centre for Research in Nanotechnology and Science, IIT Bombay, Powai, 400076 Mumbai, India; 2 Department of Electrical Engineering, IIT Bombay, Powai, 400076 Mumbai, India; 3 Department of Condensed Matter Physics and Material Sciences, TIFR, Homi Bhaba Road, 400005 Mumbai, India

## Abstract

In this article we present a three-dimensional loop Yagi-Uda array for efficient, polarization independent and directional absorption of THz radiation over a narrow frequency range (f_0_ = 0.657 THz & Q factor = 7.5). Unit cell of the array consists of three vertically stacked gold micro rings separated from each other by 30 µm thick SU-8 layers. The proposed array also exhibits a filtering response in its transmittance spectrum. The characteristics are explained by plasmon hybridization method. The transmission, reflection and absorption spectra of the structure are measured and they show a good agreement with corresponding simulated results.

## Introduction

Metamaterials are the periodic array of subwavelength composite engineered structures that possess different exotic electromagnetic properties like negative refractive index^1^, invisibility cloaking^2,3^, electromagnetically induced transparency^4,5^ and absorbance^6,7^. Recently in Tera-Hertz (THz) regime (0.1–10 THz) metamaterial absorbers have attracted attention for their compactness and perfect absorption. They have potential applications in the field of non-invasive sensing, thermal and biomedical imaging, food safety, anti-reflection coatings and spectroscopy^8–10^. In recent years different narrowband^11–13^, multiband^14–16^ and broadband^17–20^ THz metamaterial absorbers have been reported. Different physical mechanisms to explain the working principle of these absorbers are, trapping waves in isotropic Mie resonators^21^, combination of out of phase currents and non-zero order Bragg modes^22^, standing wave resonances in metal-insulator-metal structure^23^, destructive interference of multiple reflections from stacked resonators and Perfect Electrical Conductor (PEC) background^24^. A THz metamaterial based on free space impedance matching through independent tuning of its electrical and magnetic resonances was reported by Hu Tao *et al*.^25^. Different microwave receiving antennae are also designed to match their effective impedance with free space impedance for efficient absorption of the radiation at a desired frequency.

Yagi Uda antenna is a well established candidate to radiate directional beam when operated in transmitting mode and can also be used as a directional absorber while in receiving mode. It has been extensively studied in the frequency ranges covering microwave and millimeter waves, both in metallic wires^26,27^ and printed circuit form^28–31^. Towards higher frequency range like Infrared (IR) and optical regime, design of Yagi-Uda has been adopted from their radio frequency counterpart. Kosako *et al*. revisited the iconic design of Yagi Uda in nanometer scale to operate in visible frequency range^32^. In Near- Infrared (NIR) frequency range 3D Yagi-Uda antenna was reported by Daniel Dregely *et al*.^33^, which consists of three parallel linear dipole elements embedded in polymeric matrix. It was demonstrated that just like its microwave counterpart, 3-D Yagi-Uda nanostructure can be used as an efficient absorber albeit polarization dependent when illuminated by a NIR source. In THz regime, Ding *et al*. reported the simulation studies of polarization independent metamaterial broadband stop filter using a stack of identical resonators^34^.

**Figure 1:**
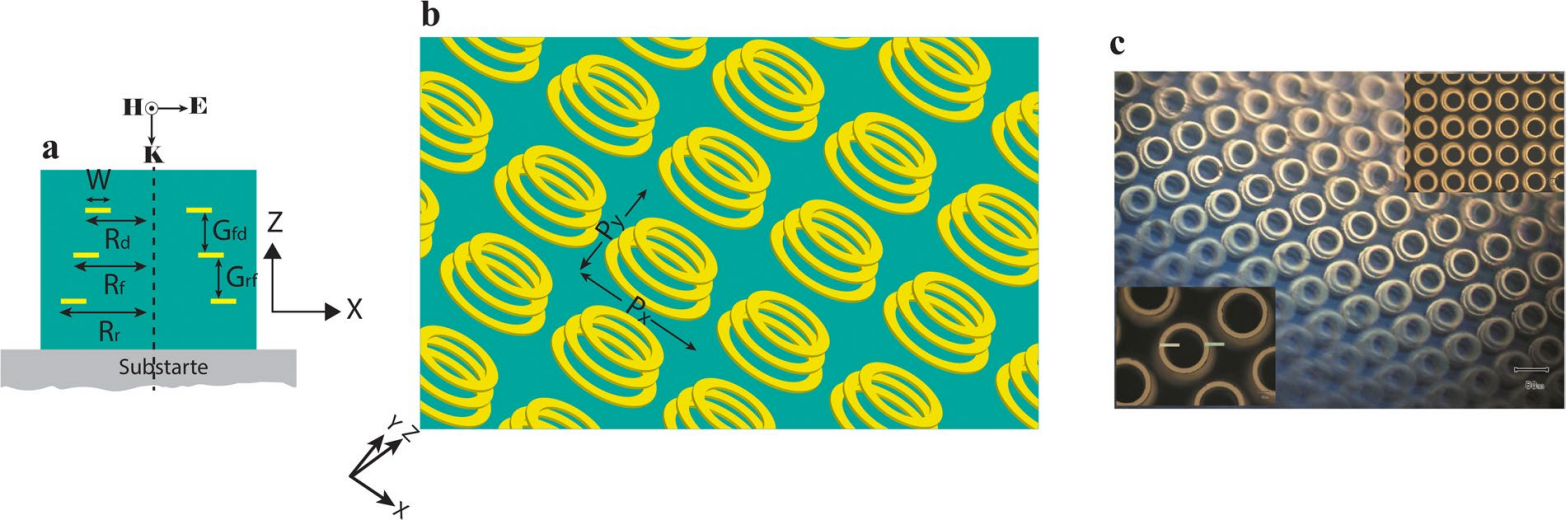
(**a**) Single unit of Yagi-Uda (side view). (**b**) Yagi-Uda array. (**c**) Optical microscope image of fabricated sample (inset: front views).

In this work, we present antenna comprised of a stack of three circular ring elements of decreasing radii as shown in Fig. 1(a). The localized plasmon mode in a circular ring is a symmetric dipole mode. Thus, the working principle of loop Yagi-Uda structure is exactly same as their linear counterpart. The only difference is that due to structural symmetry of the circular ring in both transverse directions (X and Y), the proposed structure can resonantly couple with incident wave of any arbitrary polarization. It possesses polarization independent resonant narrow band and directional absorption property in THz regime in addition to exhibiting band-stop filtering property in the transmittance spectrum. We explain the optical far-field property of THz Yagi-Uda structure using plasmon hybridization method^35^, which is analogous to the hybridization of wavefunctions in molecular orbital theory. This intuitive approach has been successfully applied to understand the optical properties of various complex nanostructures in IR and visible frequency range^36–41^.

## Results

### Schematic Description

Figure 1 illustrates the schematic description of proposed Yagi-Uda structure showing the side view of a unit cell in Fig. 1(a) and the full array of the structure in Fig. 1(b). Each unit cell of the array (Fig. 1(a)) consists of a stack of three gold rings each of thickness 0.2 µm with a 30-µm thick SU-8 layer separating two consecutive rings. In Yagi-Uda antenna terminology, from top to bottom, the three ring elements are called as director, feed (or driven element) and reflector, respectively. The outer radii (R_d_, R_f_ and R_r_) of these three ring elements are chosen to be 43, 47 and 54 µm, respectively. The width (w) of each ring element is considered to be 10 µm. The complex permittivity of gold in THz regime is described by Drude model with plasma frequency ω_p_ = 1.366e16 rad/sec and collision frequency ω_c_ = 5.648e13 rad/sec^42^. The THz permittivity and loss tangent of SU-8 are taken as 2.92 and 0.055, respectively^43^. The periodicity of the array (P_x_ and P_y_) is chosen to be 130 µm in both x and y- directions (Fig. 1(b)).

A square array of Yagi-Uda structure of area 5 mm × 5 mm was fabricated on a 1 mm thick THz-transparent fused-silica substrate with standard optical lithography process (for details see method). Figure 1(c) shows the optical microscope image of the fabricated structure along with zoomed in regions as insets.

### Numerical simulation and experimental verification

**Figure 2:**
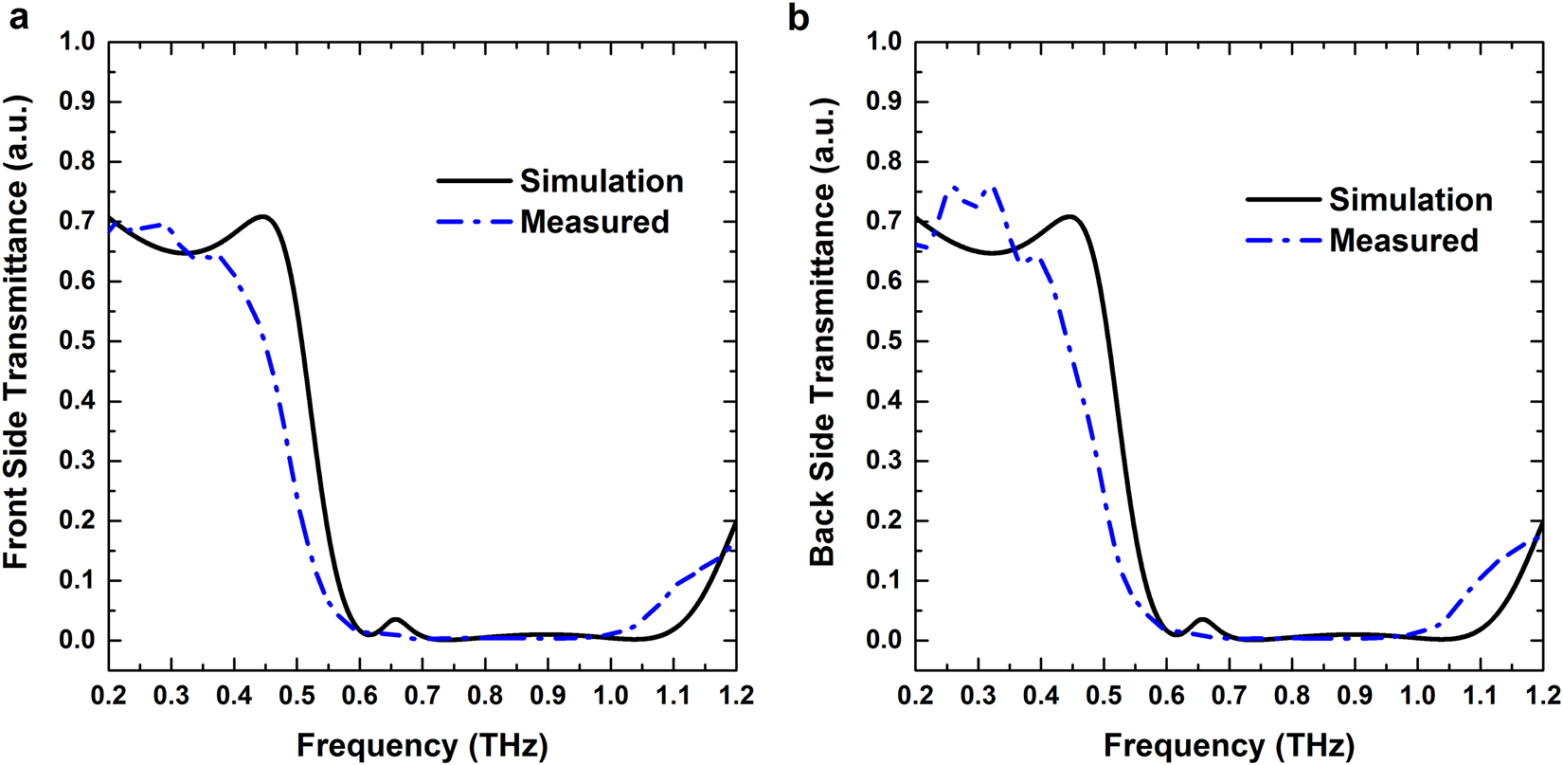
Simulated and measured transmission spectra for (**a**) Front side and (**b**) Back side illumination.

**Figure 3:**
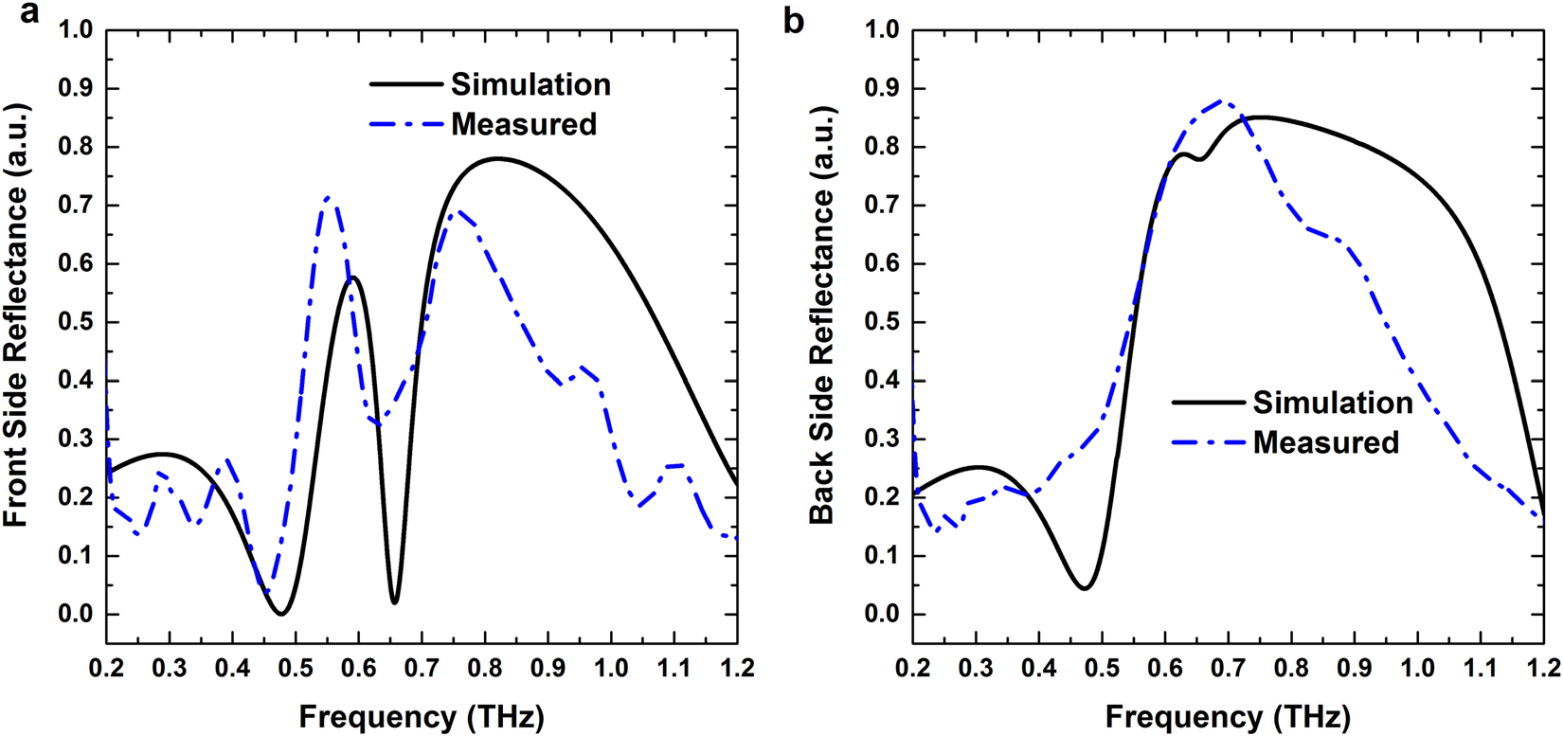
Simulated and measured reflection spectra for (**a**) Front side and (**b**) Back side illumination.

**Figure 4:**
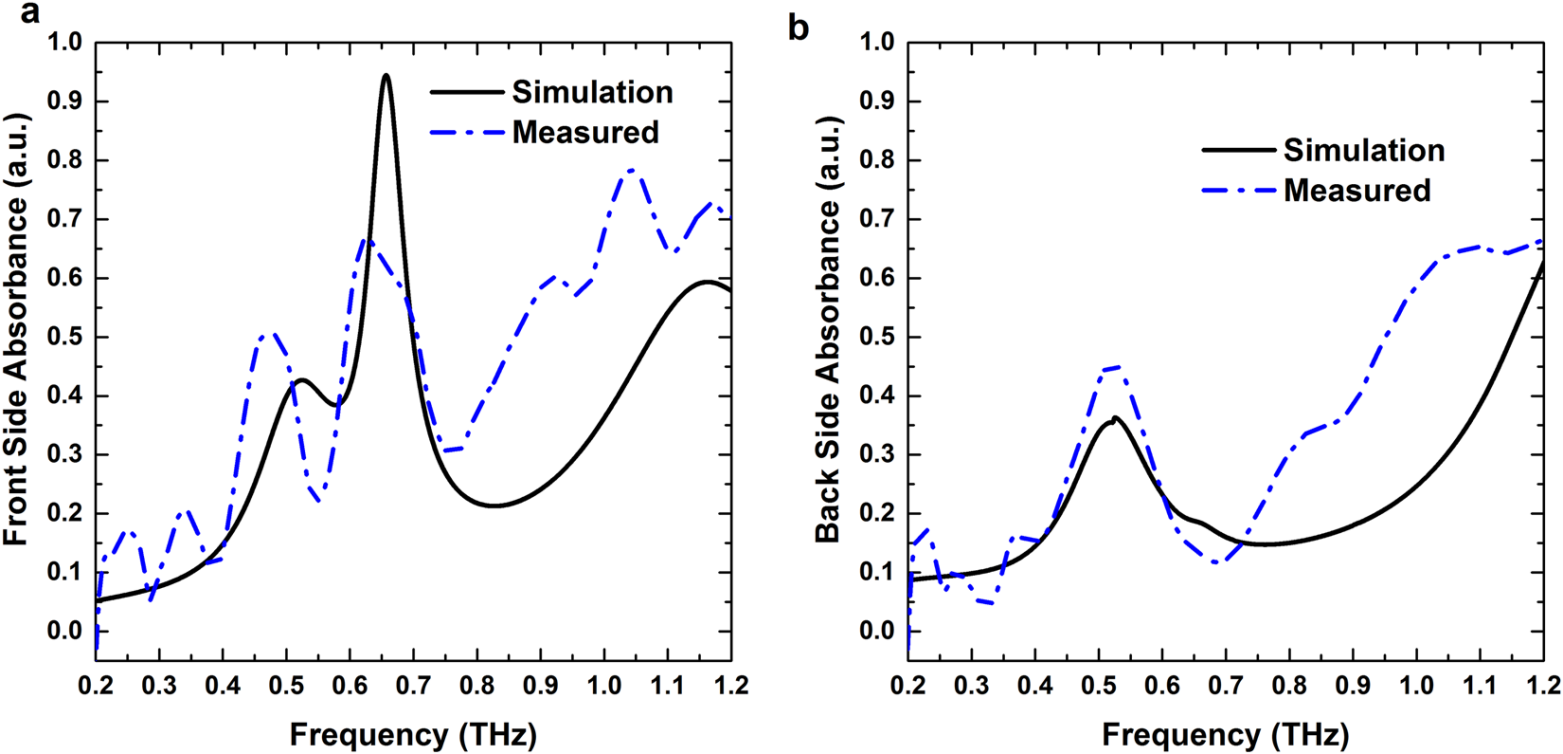
Simulated and measured absorption spectra for (**a**) Front side and (**b**) Back side illumination.

THz far field properties of the Yagi-Uda array were numerically simulated followed by the experimental verifications of simulated results. All simulations were performed using CST Microwave studio. A normal incident x-polarized plane wave is considered throughout the numerical studies (shown in Fig. 1(a)). THz pulse impinging on the structure from director (reflector) side is indicated as front (back) side illumination. In our THz-Time Domain Spectroscopy (THz-TDS) measurements, we measured transmission and reflection for both front and backside illumination. All the simulated results with their corresponding experimental verifications are shown in Figs 2–4. Figure 2(a) and (b) show that the transmission spectra for both front and back side illumination are same. This indicates the transmittance through the structure is direction-independent of the incoming radiation.

Figure 3(a) and (b) show that the front side reflection of the structure is different from the back side reflection. This is due to the symmetry breaking of the structure in the line of wave propagation. The resonant dip at 0.657 THz in the front side reflectance is absent in case of the back side reflectance spectrum. Therefore, the Yagi-Uda array acts as a resonant absorber when THz wave is incident from director side of the structure.

We calculate the absorbance spectra from $A=1-(T+R)$ and plot in Fig. 4(a) and (b), respectively. The expected resonant peak in the front side absorbance spectrum is noticed at 0.657 THz where both the transmittance and reflectance are minimal. On the other hand, for back side illumination there is no significant absorbance peak as most of the field is reflected from the structure. The red shift of the measured absorption peaks with respect to the corresponding simulated peaks could be attributed to the slight mismatch in the material parameters and dimensions used in simulations and the actual.

**Figure 5:**
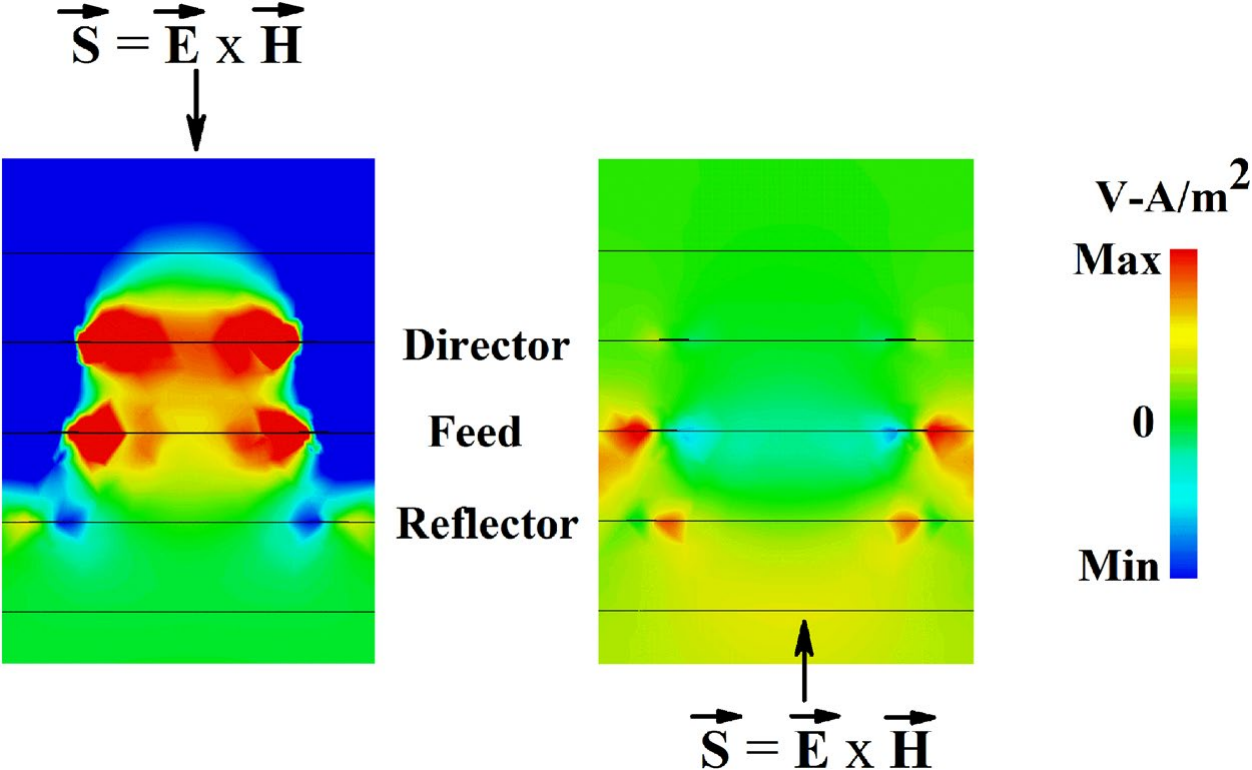
Power flow in the structure for front side (left) and back side illumination (right).

We plot power flow at the absorption peak (0.657 THz) for both front and back side illumination in Fig. 5. From the figure it is noticed that maximum power is absorbed near the feed and director when THz pulse is impinged on the structure from director side. Whereas, less power is absorbed when wave is coming from the reflector side. The angle dependent absorbance of the array at resonant absorption peak (0.657 THz) and two half power points (0.61 and 0.7 THz) are also plotted in Fig. S1 (Supplement). As shown in the figure, power absorption ability is higher when THz wave is impinging on the structure from the director side rather than the reflector side.

## Discussion

**Figure 6:**
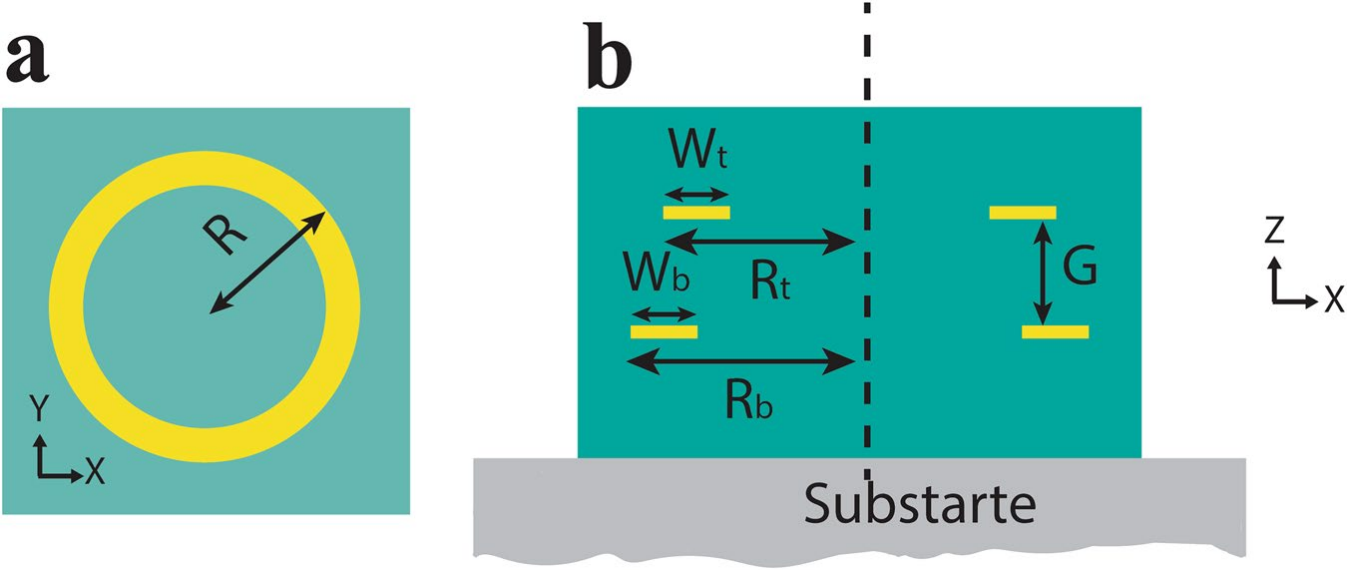
(**a**) Single ring (front view). (**b**) Single unit of double stacked rings (side view).

To elucidate the far-field optical properties of the proposed Yagi-Uda structure a detailed study of plasmon hybridization scheme is presented in this section. We start our discussion with single-layer ring array. The unit cell (top view) is shown in Fig. 6(a).

The lowest energy plasmon mode in a circular ring is same as that in an infinitely long cylindrical rod where the guided wavelength of the plasmon is equal to the circumference of the ring (Fig. S2)^44,45^. Simulated transmittance and reflectance spectra of the circular ring array for three different ring radii are shown in Fig. S3 (a). One resonant dip noticed in each of the transmittance spectra attributes to plasmon resonance that arises from dipole oscillation of charges inside the ring. The Z-component of the electric field at the bottom surface of the ring (shown in Fig. S3 (b)) confirms dipole oscillation.

After single ring study, we studied an array of double stacked rings. The structure is fully embedded in SU-8. The vertical gap (G in Fig. 6(b)) between the two rings is chosen to be 30 µm, which is same as that between two consecutive ring elements in the final Yagi-Uda structure (G_rf_ and G_fd_ in Fig. 1(a)). To understand the coupling effect of two consecutive rings in the Yagi-Uda structure we consider two different sets for top and bottom rings. Outer radii of top and bottom rings are as follows (a) 47 and 54 µm, respectively (feed and reflector) and (b) 43 and 47 µm, respectively (director and feed). The plasmon hybridization method for the two ring structure along with the simulated transmitted and reflectance spectra are provided in supplementary section (Figs S4–S6).

Here we present the detailed study on the full Yagi-Uda array whose unit cell consists of three stacked ring elements (Fig. 1(a)). For easy visualization of transmission minima we plot transmission coefficient as a function of frequency for the three ring stack structure in Fig. S7 (solid black curve). The three resonant transmission minima at 0.615 (I), 0.74 THz (II) and 1.02 THz (IV) are indicated in the figure.

**Figure 7:**
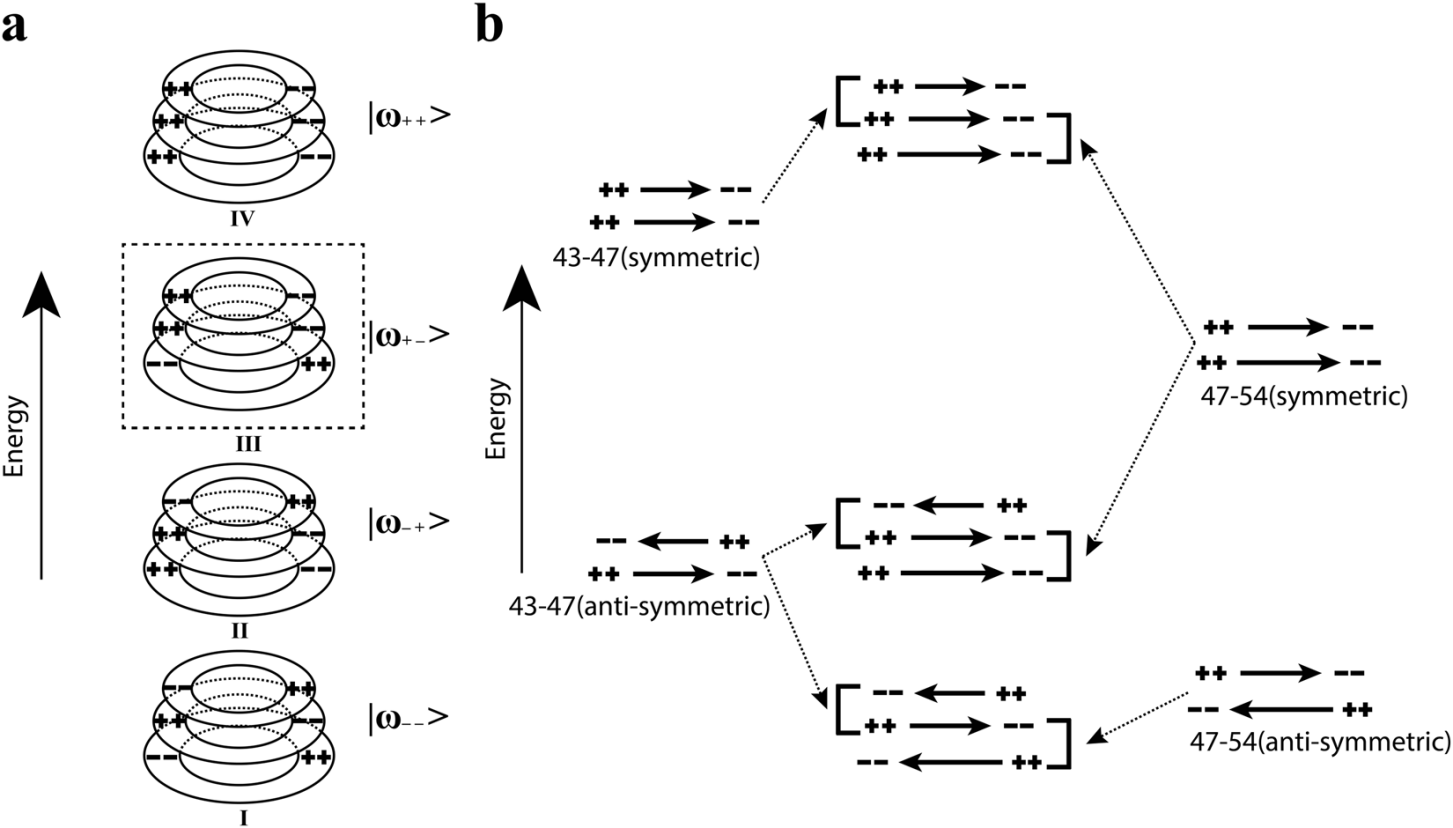
(**a**) Hybridized modes of three stacked rings. (**b**) Each mode consists of two sub-hybridized modes. Each of the hybridized modes is labelled based on which of the two rings (of radius 43, 47 and 54 μm) are contributing.

Formation of different hybridized modes from bare plasmon interactions of three rings are shown in Fig. 7(a) with the resonances labelled Ito IV. Lowest energy plasmon $|{\omega }_{--}\rangle$ corresponds to the out of phase charge oscillation inside each ring with respect to its adjacent rings. Charge oscillations inside three rings are all in phase in case of highest energy plasmon mode $|{\omega }_{++}\rangle$. Lower energy intermediate mode $|{\omega }_{-+}\rangle$ is generated when charge oscillations in the top two rings are out of phase with respect to each other but charge oscillations in bottom two rings are in the same phase.

Higher energy intermediate plasmon $|{\omega }_{+-}\rangle$ cannot form in this structure. The reason is as follows: in each hybridized mode of three stacked rings, two sub-hybridized modes coexist, as illustrated in Fig. 7(b). One sub-hybridized mode from the top pair of rings and another from the bottom pair of rings with common middle ring. In case of higher energy intermediate plasmon $|{\omega }_{+-}\rangle$ these two sub-hybridized modes are top pair symmetric and bottom pair antisymmetric plasmon modes. The plasmon resonances for these two modes are 0.62 THz (**I**) and 1 THz (**II’**), respectively, as marked in Fig. S4 in supplementary section. Due to this large energy separation between the two sub-hybridized modes, $|{\omega }_{+-}\rangle$ cannot be generated^35^.

**Figure 8:**
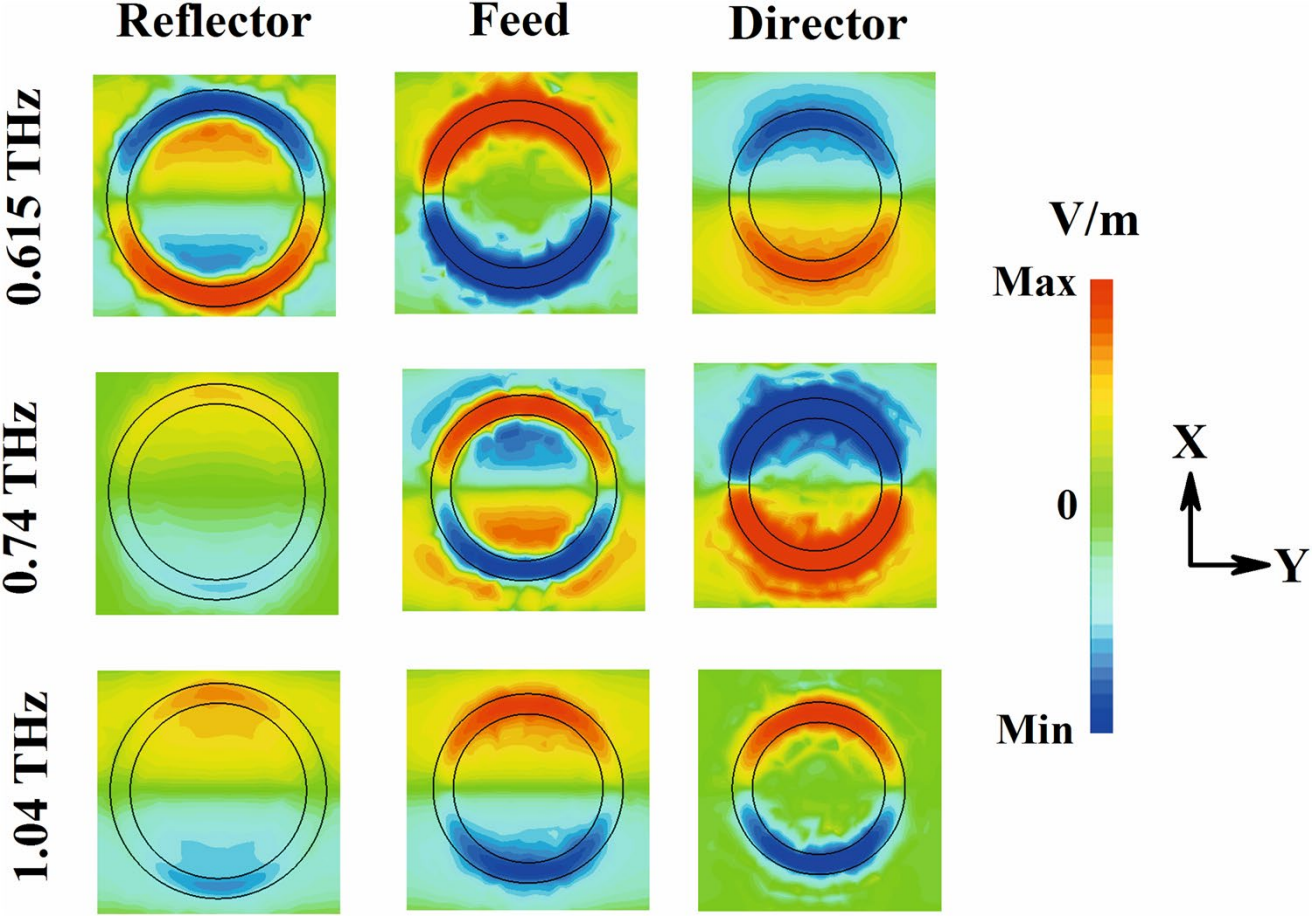
Z-component of electric field at bottom surfaces of three rings; Reflector (bottom), feed (middle) and director (top).

The Z-components of electric field distribution at bottom surfaces of three rings at different resonant minima are shown in Fig. 8. These also support the formation of different hybridized plasmon modes at their corresponding resonances. At lowest energy plasmon frequency (0.615 THz) as the charge oscillation in the three rings are out of phase with respect to each other, two oppositely polarized magnetic plasmons are formed inside the two spacer layers, respectively, as shown in Fig. S8. Similarly, at 0.74 THz one magnetic plasmon is formed inside the spacer layer between the top two rings.

As observed in the transmittance spectrum of Yagi-Uda structure (Fig. 2(a); solid black curve), successive transmittance minima originating from different hybridized plasmon modes are merged and form a continuous broad stop band (from 0.6 THz to 1.1 THz), where the transmission through the structure is almost zero^46^. So the Yagi-Uda array can be used as a band stop filter to suppress the transmission over a broad frequency range.

**Figure 9:**
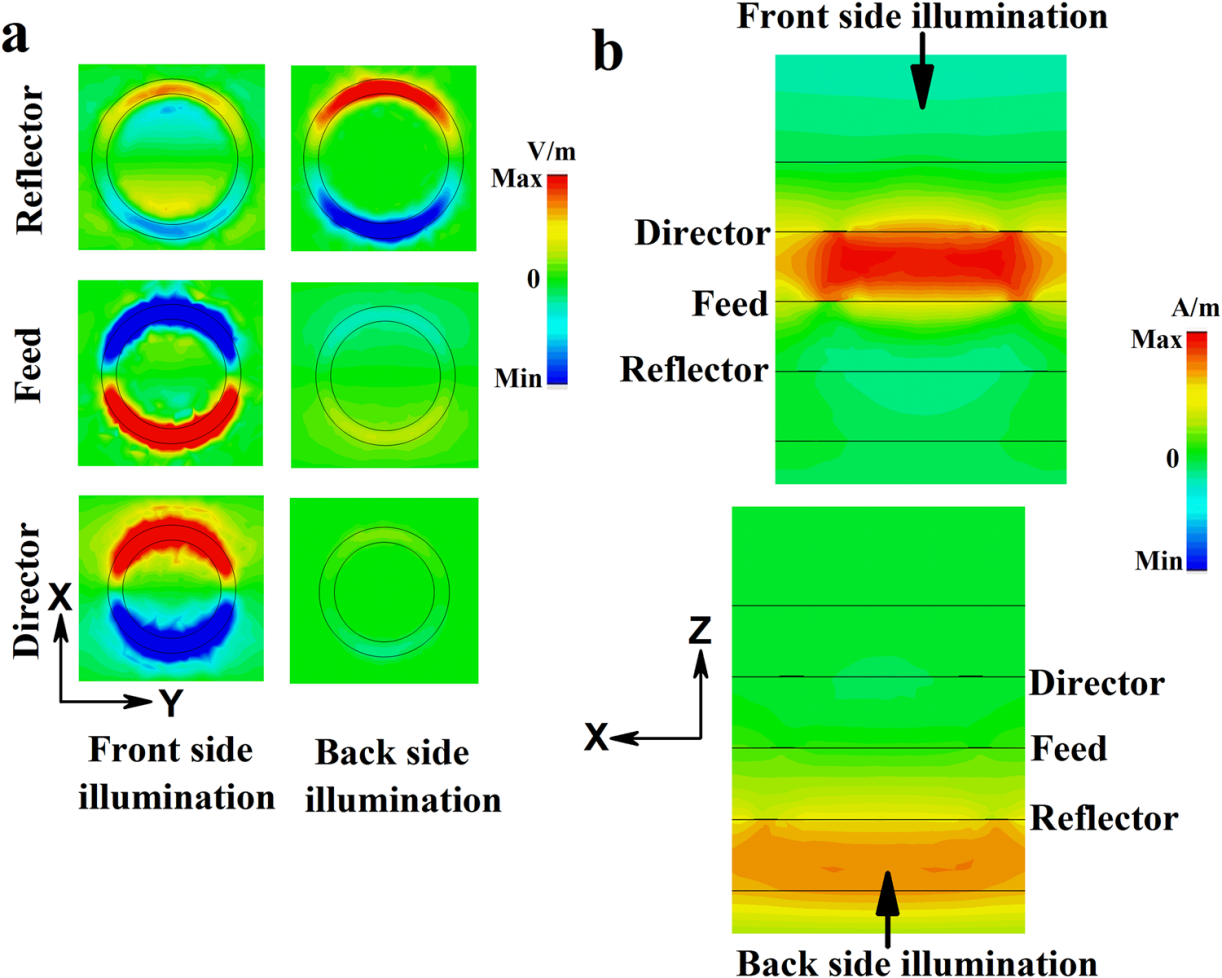
(**a**) Z-component of the electric field at bottom surfaces of three rings; Reflector (bottom), feed (middle) and director (top). (**b**) Y-component of magnetic field inside dielectric spacer layers for both front and back side illumination at 0.657 THz.

At 0.657 THz, a significant difference is observed between the reflections from the structure for the front and back side illuminations, respectively. The reflector of radius 54 µm has a reflection peak (0.67 THz) close to the frequency of 0.657 THz as shown in Fig. S3 (black curve with triangular marks). Therefore, at this frequency THz wave impinging from reflector side is directly coupled to the reflector first and generate a bright dipolar resonance, which is shown in Fig. 9(a) (scale bar for the Z-component of electric field is shown in right panel). The other two resonators are shielded behind the reflector thus weakly coupled with the wave. This leads to a high reflection from the structure for back side illumination at 0.657 THz that is predominantly contributed by the reflector element. On the other hand, for front side illumination the impinging wave at 0.657 THz can see the complete three stacked ring structure. As shown in Fig. 9(a) left panel the director and feed elements are strongly coupled to the incoming wave. Due to out of phase charge oscillations inside the director and feed, a strong magnetic response is generated between them. Therefore, both the electric and magnetic field of the incoming wave are trapped inside the structure at 0.657 THz. At this frequency the impedance of the structure (Z = √ (µ/ε)) matches with free space impedance (µ = ε) for front side illumination. Therefore, reflectance reduces to zero (Fig. 3(a); solid black curve) and this results in high resonant absorption at this frequency (Fig. 4(a); solid black curve).

## Conclusion

In summary, we have demonstrated a loop Yagi-Uda absorber in THz, which is consisting of three stacked micro rings of decreasing radii from bottom to top. We have explained its optical property using plasmon-hybridization method. It exhibits resonant directional absorbance property with absorption amplitude higher than 90%. The measured transmssion, reflection and absorption spectra are found to be well matched with the corresponding simulated results. The proposed structure can be used as a band reject filter to suppress the transmission from 0.2 to 1.1 THz.

## Methods

### Sample Fabrication

A square array of Yagi-Uda structure of area 5 mm by 5 mm was fabricated on a 1 mm thick THz-transparent fused-silica substrate. A 30 µm thick SU 8–2025 layer was spin coated on the substrate and was first soft baked at 65 °C and then at 95 °C for 3 and 5 minutes, respectively (Pre-exposure bake). Then soft baked SU8 was exposed to 365 nm wavelength UV light through a 5 mm × 5 mm window of an iron oxide mask followed by a post exposure bake at 65 °C and 95 °C for 1 and 6 minutes, respectively. Pre and post-exposure baking was performed to dry the film and to reduce internal stress in it, respectively. Sample was then immersed in SU8 developer, followed by rinsing in IPA and then dry cleaned by pressurized dry nitrogen gas. A final hard baking at 150 °C for 15 minutes was carried out to make the SU8 extremely hard. A single layer PPR (S-1813) was spin coated onto the hard baked SU8 layer and solvent was evaporated by baking it at 90 °C for 4 minutes. Image of micro-ring structures of the reflector layer are then transferred from the iron oxide mask to PPR layer by standard optical lithography technique and subsequent development of UV exposed PPR by MF-319 developer, followed by rinsing in DI water and then dry cleaned by pressurized pure nitrogen gas. After that Ti (5 nm)-Au (190 nm) was sputtered and subsequently lift-off was performed to remove the remaining resist. The whole procedure was repeated two more times to create subsequent driver and director array as shown in Fig. 1(c) and (d). It was ensured that subsequent layers were made exactly on top of their corresponding bottom layers by gold alignment marks of feature size 2 µm. One final SU8 layer was deposited to embed the whole structure in SU8 matrix.

### THz-TDS Spectroscopy

Transmittance and reflectance of the fabricated sample was measured using THz-Time Domain Spectroscopy (THz-TDS). THz-pump pulse was generated by dipole photoconductive antenna fabricated on a Low temperature Gallium Arsenide (LT-GaAs) substrate (commercial BATOP source) by a 10 femtosecond Ti: sapphire laser beam with 80 MHz repetition rate. A Quartz beam splitter was used to simultaneously measure both transmittance and reflectance in the same set up.The transmitted and reflected THz pulse was detected by electro-optic sampling process. In this process, 800 nm optical probe pulse and transmitted (or reflected) THz signal were together impinged on a Zn-Te (Zinc-Telluride) crystal followed onto a quarter-wave plate and a Wollaston prism. After that, the probe pulse was sent to a balanced photodiode with rise time of 100 nanosecond whose output is recorded by a lock in amplifier (SR-865). The measurement was performed at room temperature. A 1 mm fused silica substrate was used as the reference for transmittance and a 0.2 µm thick gold mirror was used as the reference for reflectance. The recorded time domain signal was amplitude against the time delay between pump and probe pulses. We repeated the experiment three times and performed FFT (Fast Fourier Transform) on them. The averaged Fourier transform signal is normalized with respect to the corresponding reference samples.

## Electronic supplementary material

Supplementary Information
