# Supplementary material for: Study of THz-Plasmon hybridization of a loop Yagi-Uda absorber
Source: Sci Rep. 2017 Dec 5;7:16961. doi: 10.1038/s41598-017-17311-3 (PMC5717262; doi:10.1038/s41598-017-17311-3)
Supplement: Supplementary file 1 — Supplementary Information [file 41598_2017_17311_MOESM1_ESM.pdf]

Study of THz-Plasmon hybridization of a loop Yagi-Uda absorber

Arnab Pattanayak[1,*], Sandipta Roy[1,+], Goutam Rana[2,+], Siddhartha P.Duttagupta[1,2], Venu Gopal Achanta[3], S.S. Prabhu[3]

[1]Centre for Research in Nanotechnology and Science, IIT Bombay, Powai, 40076, Mumbai, India
[2] Department of Electrical Engineering, IIT Bombay, Powai, 40076, Mumbai, India
[3] Department of Condensed Matter Physics and Material Sciences, TIFR, Homi Bhaba Road, 40005, Mumbai, India
[*]arnab1134@gmail.com
[+]these authors contributed equally to this work

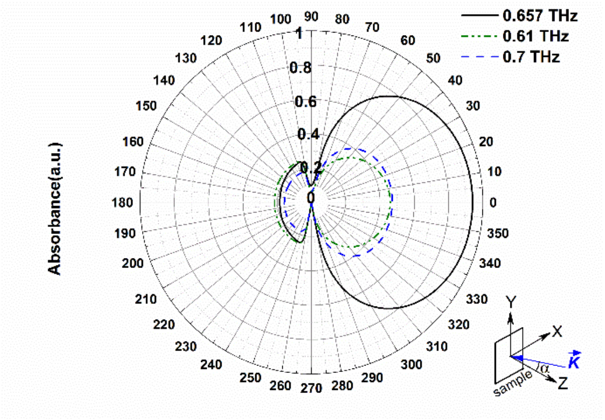

Supplementary Figure S1. Polar plot of absorbance as a function of incident angle of incoming wave. Angle between propagation vector of impinging wave and normal axis of the sample is varied as shown in the bottom right corner of Fig. S1. The plot indicates that the absorption in the structure is strongly dependent on the direction of incoming radiation.

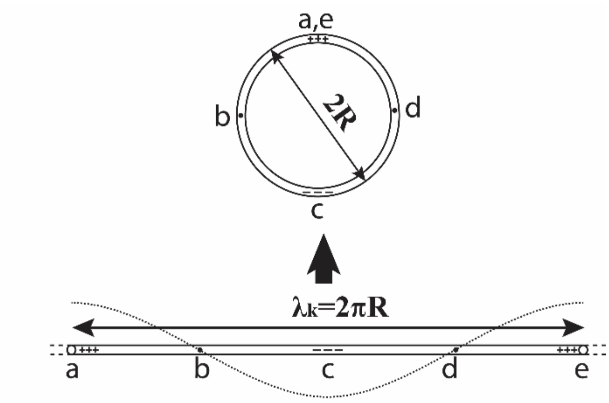

Supplementary Figure S2. Lowest energy plasmon mode in circular ring; the dotted curve on thin infinite cylinder represents sinusoidal charge distribution. Guided wavelength (λ_k , K= propagation

constant in the medium = ω/v, v= wave velocity in medium) depends on the permittivity and thickness of the medium.

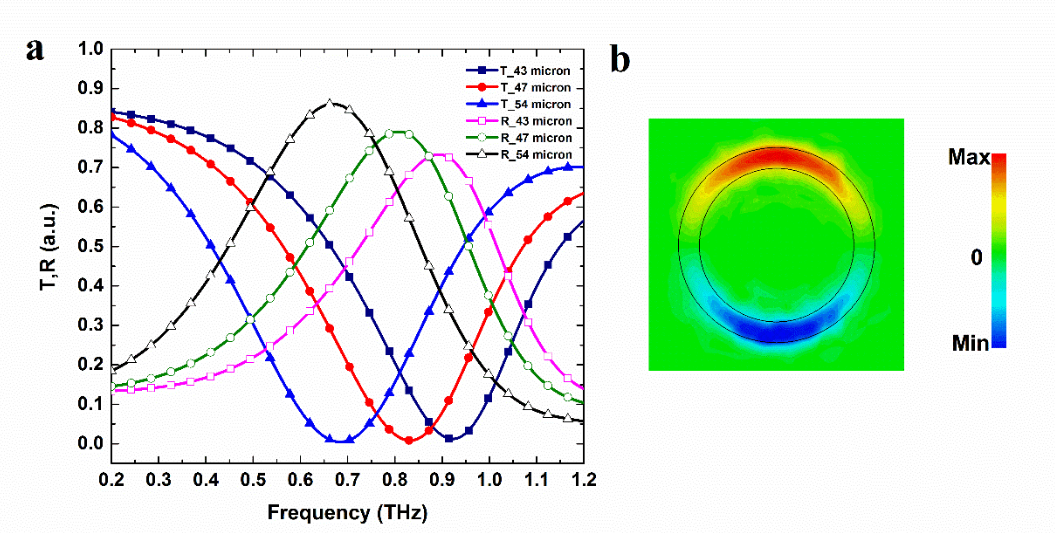

Supplementary Figure S3. (a) Transmittance and reflectance spectra of single ring arrays. Each transmittance spectrum has one resonance dip due to dipole oscillation of charges inside the ring. (b) **Z-component of electric field distribution at bottom surface of ring.** This confirms the dipole nature of charge oscillation.

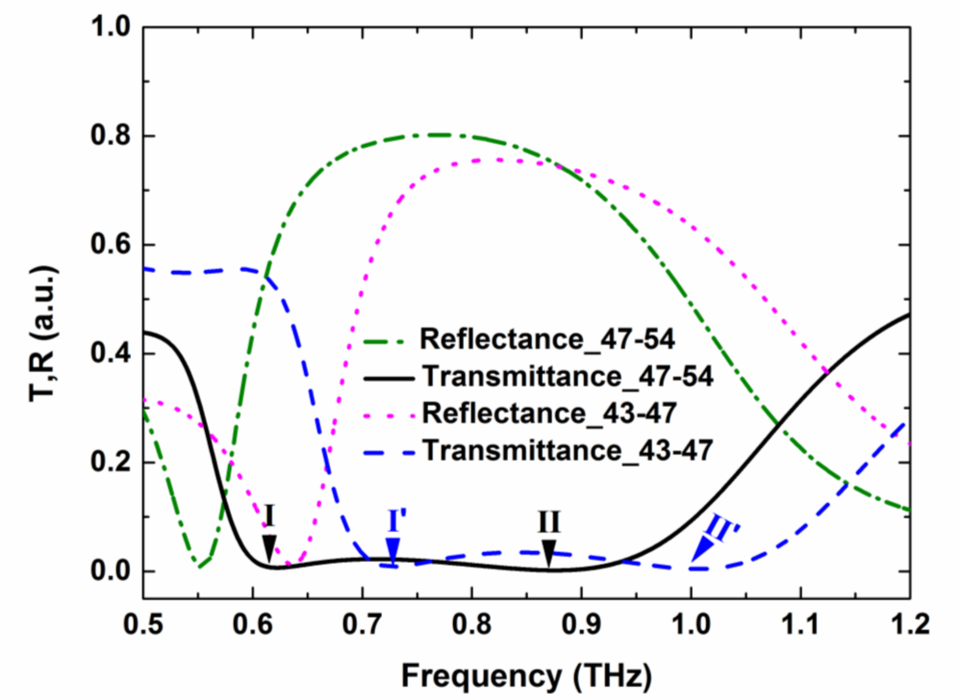

Supplementary Figure S4. Transmittance and reflectance spectra of double stacked rings for set a and b. Instead of one resonance dip in the transmittance spectrum as seen for single layer ring array (Fig. S3 (a)), transmittance spectrum of double ring array shows for set (a) ((b)) two resonant minima,

antisymmetric mode at 0.62 THz (0.73 THz) denoted by **I** (**I'**) and symmetric mode at 0.87 THz (1THz) denoted by **II** (**II'**). Plasmon hybridization method shown in Fig. S5 is employed to understand the origin of these two transmission minima[36].

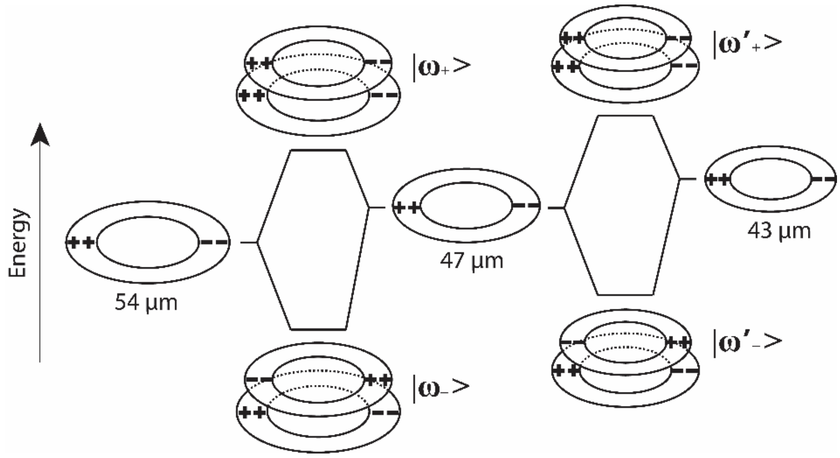

Supplementary Figure S5. Plasmon hybridization of double stacked rings of different radii. The primitive ring plasmons of two closely spaced rings interact with each other resulting in splitting of the bare plasmon resonances into two new resonances: higher energy symmetric plasmon mode $|\omega_+\rangle$ which corresponds to higher resonance 0.87 THz (marked as **II** in simulated transmittance spectra) and lower energy antisymmetric plasmon mode $|\omega_-\rangle$ which corresponds to lower resonance 0.62 THz (marked as **I**). The symmetric and antisymmetric plasmon modes originate from the in- and out- of phase charge oscillations inside the top and bottom rings, respectively. In case of symmetric plasmon mode, as the charges of same polarity coincide in top and bottom rings during oscillations, they repel each other. As a result, the restoring force of charge oscillation increases which leads to a higher resonance plasmon than that of single layer ring array. In case of antisymmetric plasmon, attraction between charges of opposite polarity in the top and bottom rings cause reduction in the restoring force. This results in lower resonance compared to bare plasmon of single ring. E_z components at the bottom surfaces of two rings confirm the fact of hybridization of electric plasmon polariton. As seen in Fig. S5 (a), for lower antisymmetric (higher symmetric) plasmon resonance, the induced E_z components of electric field in the top and bottom rings are opposite (same) in phase. At lower antisymmetric resonance, a circular current loop is formed inside the dielectric spacer layer between the two rings resulting in a magnetic

dipole oscillation as depicted in Fig. S5 (b). This magnetic polariton interacts with magnetic field of incoming wave, so a strong magnetic response is associated with antisymmetric resonance.

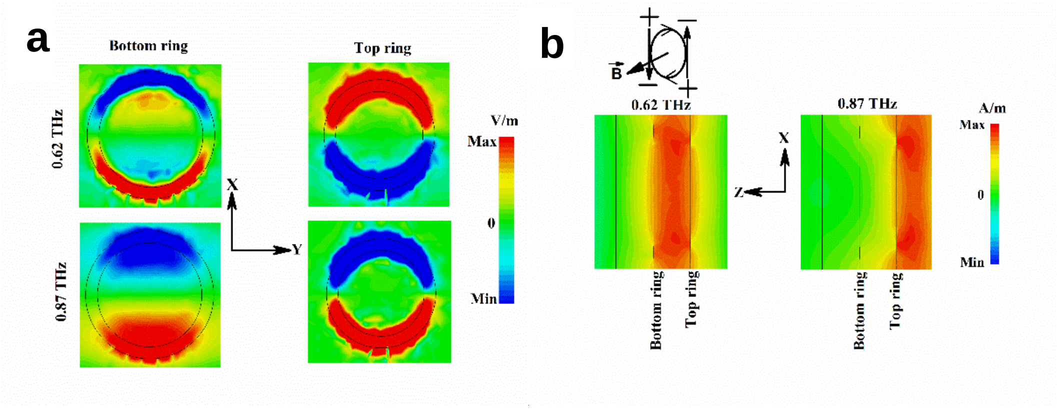

Supplementary Figure S6. (a) **Z-component of electric field at the bottom surface of bottom and top rings.** For lower frequency anti-symmetric mode (0.62 THz) dipole oscillations of charges are out of phase, whereas for higher frequency symmetric mode (0.87 THz) dipole oscillation of charges are in phase (b) **Y-component of magnetic field inside dielectric spacer layer.** For anti-symmetric mode a circular current loop is formed generating a magnetic dipole oscillation in the spacer layer between two rings.

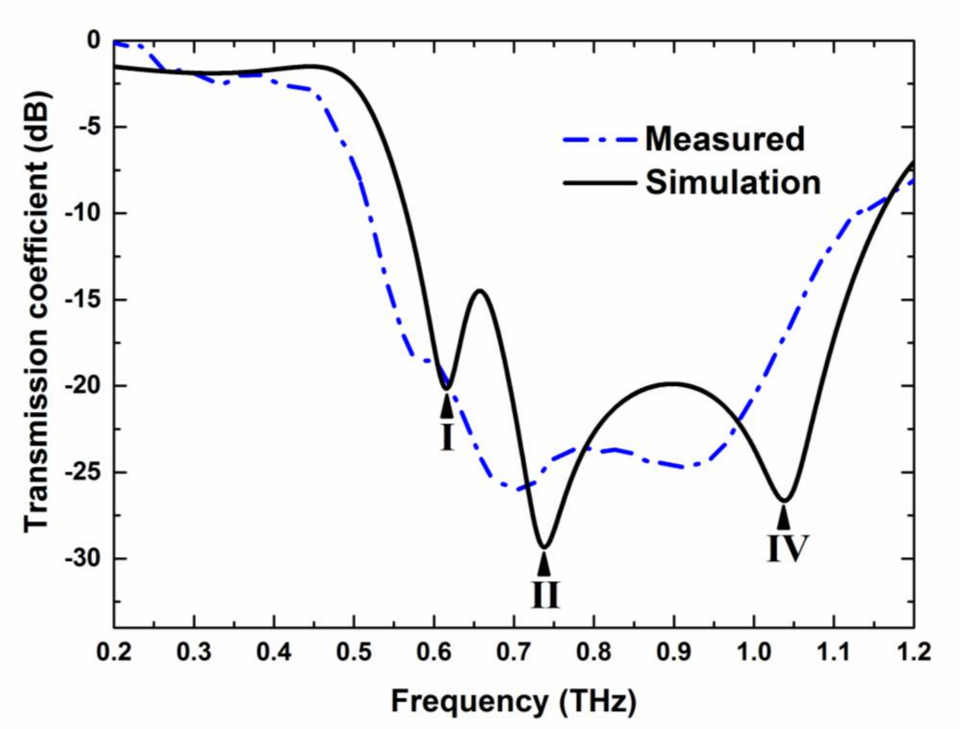

Supplementary Figure S7. Transmission coefficient. Transmission coefficient =10log$_{10}$ (Transmittance).Three resonant minima are seen in the simulated transmission coefficient curve (solid black curve). Origin of these transmission minima are explained using plasmon hybridization method (Main text). The red shift in the measured spectrum (dash-dotted blue curve) is due to the slight mismatch in the material parameters and dimensions used in simulations and actual.

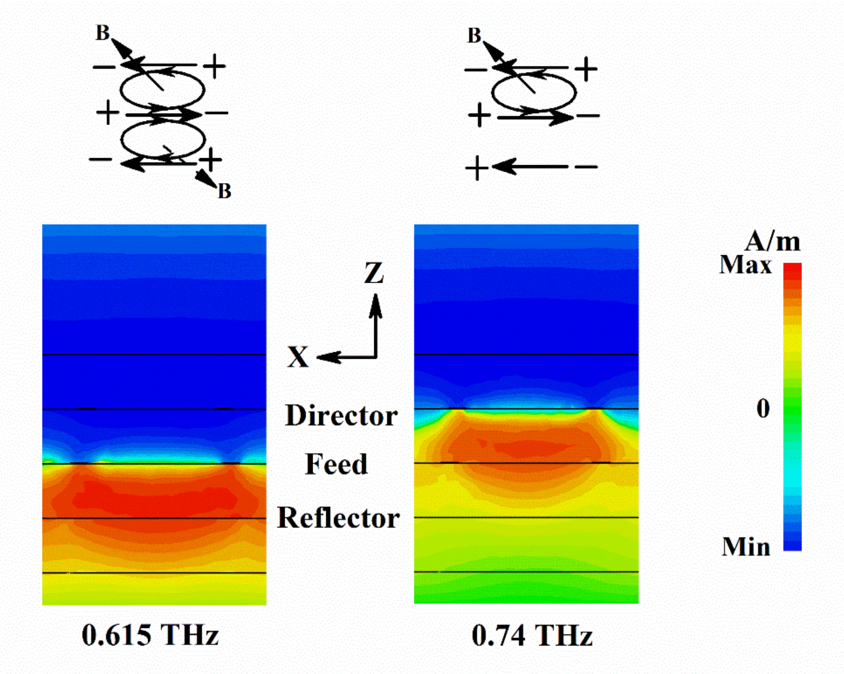

Supplementary Figure S8. Y-component of magnetic field inside dielectric spacer layers. At 0.615 THz, charge oscillations in all three rings are out of phase with respect to each other. Between each pair of rings magnetic dipole oscillation is generated inside the spacer layer. However, these two magnetic dipole oscillations are out of phase. At 0.74 THz, between feed and director, magnetic dipole oscillation is generated.
